# Data on genome analysis of *Bacillus velezensis* LS69

**DOI:** 10.1016/j.dib.2017.04.053

**Published:** 2017-05-05

**Authors:** Guoqiang Liu, Yingying Kong, Yajing Fan, Ce Geng, Donghai Peng, Ming Sun

**Affiliations:** State Key Laboratory of Agricultural Microbiology, College of Life Science and Technology, Huazhong Agricultural University, Wuhan 430070, Hubei, People׳s Republic of China

## Abstract

The data presented in this article are related to the published entitled “Whole-genome sequencing of *Bacillus velezensis* LS69, a strain with a broad inhibitory spectrum against pathogenic bacteria” (Liu et al., 2017) [1]. Genome analysis revealed *B. velezensis* LS69 has a good potential for biocontrol and plant growth promotion. This article provides an extended analysis of the genetic islands, core genes and amylolysin loci of *B. velezensis* LS69.

## **Specifications Table**

**Table:** 

Subject area	Biology
More specific subject area	Microbiology
Type of data	Table, text file, figure
How data was acquired	Genome sequencing: HiSeq. 2500 platform (Biomarker Technologies, Beijing, China),
	Bioinformatics approaches: NCBI Prokaryotic Genomes Automatic Annotation Pipeline (PGAAP), antiSMASH (http://antismash.secondarymetabolites.org),
	BAGEL3 (http://bagel.molgenrug.nl/index.php/bagel3), IslandViewer 3 (http://www.pathogenomics.sfu.ca/islandviewer/),
	BPGA (Bacterial Pan Genome Analysis tool) (http://sourceforge.net/projects/bpgatool/), multiple alignments using Clustal Omega at EBI.
Data format	Analyzed
Experimental factors	Genome sequencing, genome annotation, active metabolites prediction, multiple alignments
Experimental features	Whole-genome sequencing of *Bacillus velezensis* LS69 was performed by using Illumina HiSeq. 2500 platform. Secondary metabolites clusters of *B. velezensis* LS69 were predicted by using antiSMASH and BAGEL3. Genetic islands in *B. velezensis* strain LS69 were predicted by using IslandViewer 3. COG distribution of the core genes, accessory genes and unique genes were analyzed by using BPGA pipeline. Blast analysis was performed to reveal the differences between amylolysin loci in *B. velezensis* LS69 and the corresponding loci in other *B. velezensis* strains.
Data source location	*Bacillus velezensis* LS69 was isolated from the rice field of Lichuan city, Hubei Province (China).
Data accessibility	The whole genome sequence of *B. velezensis* LS69 has been deposited in GenBank under the accession number CP015911.

## **Value of the data**

• *Bacillus velezensis* LS69 were found to contain an abundant of gene clusters required for synthesizing antimicrobial metabolites and promoting plant growth. Most of unique genes for strain LS69 were clustered in the seven genetic islands. Here we provided an detailed analysis of the genes on the genetic islands.
• *Bacillus velezensis* strains were known to produce versatile metabolites with antimicrobial activity and secrete a variety of compounds promoting plant-growth. Here we provided an analysis of the COG distribution of the core genes, accessory genes and the unique genes in 12 *B. velezensis* strains.
• Our data provide an extended analysis of the amylolysin cluster, which was found to be unique for the *B. velezensis* LS69 by comparative analysis with the highly homologous *B. velezensis* strains.

## 1 Data

**Fig. 1:**
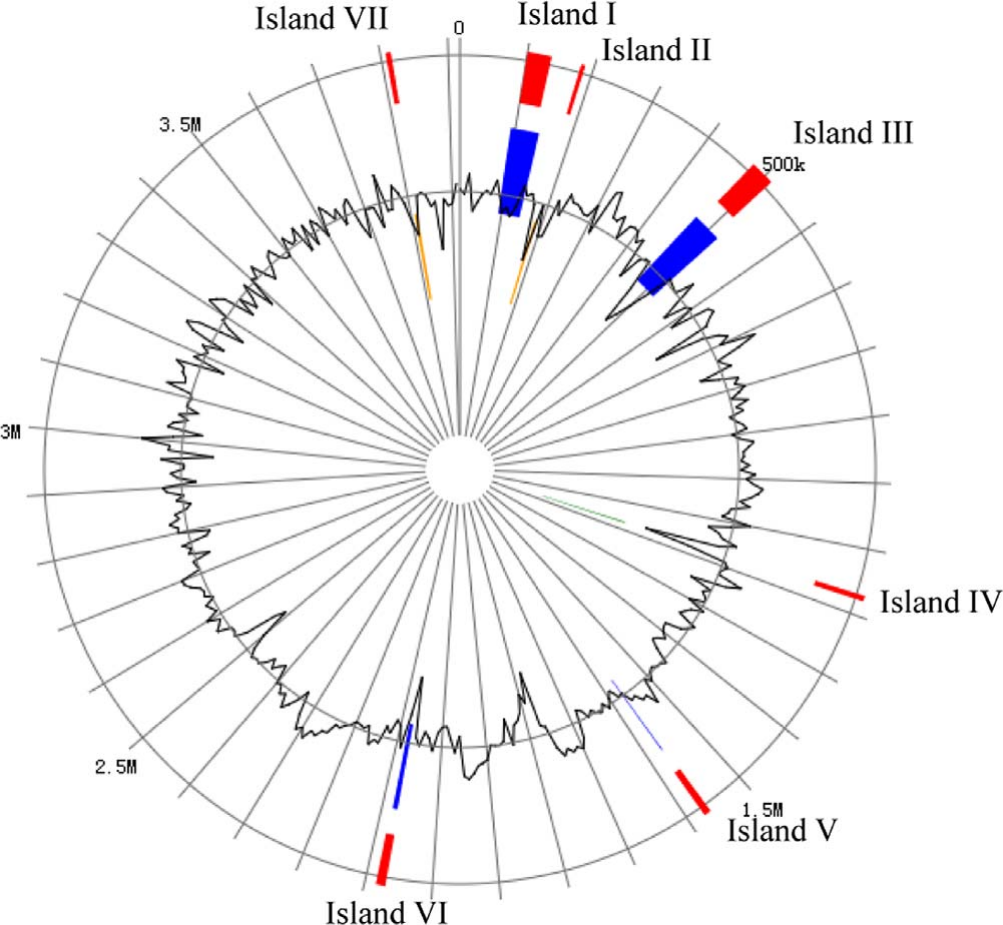
Genetic islands in *Bacillus velezensis* strain LS69. The seven genetic islands in *B. velezensis* LS69 were predicted by using Island Viewer 3. All the genes on the genetic islands were displayed in the Supplementary Table 1.

**Fig. 2:**
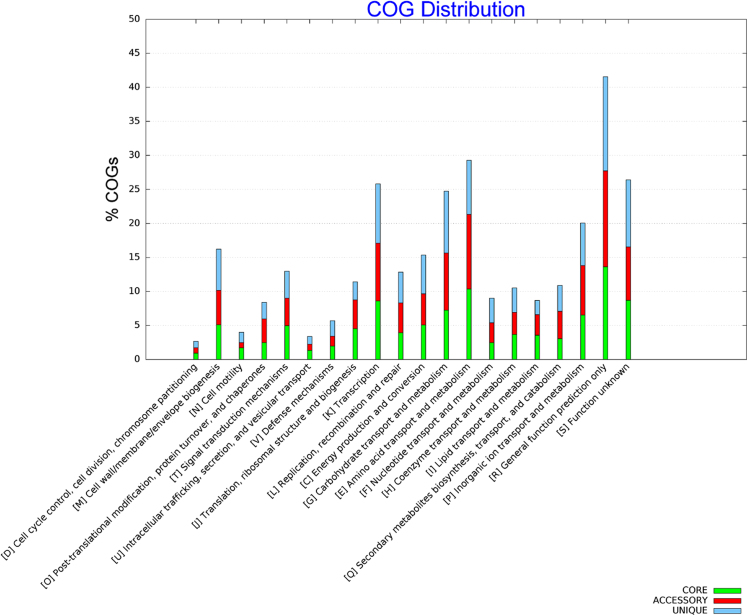
Clusters of Orthologous Groups (COG) distribution of the core genes, accessory genes and unique genes in *Bacillus velezensis* strains. BPGA pipeline was used to determine the COG distribution. A total of 12 *B. velezensis* strains were selected, including the strain LS69, CBMB205, SQR9, FZB42, YJ11-1-4, CAU B946, JJ-D34, JS25R, NAU-B3, UCMB5033, UCMB5113 and YAU B9601-Y2.

**Fig. 3:**
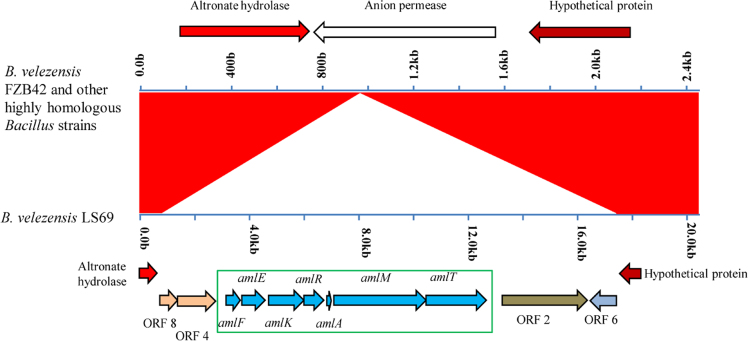
Genetic loci associated with producing amylolysin in *Bacillus velezensis* LS69. Compared with other highly homologous *B. velezensis* strains, one 16 kb fragment was found to be unique for the strain LS69. This genetic loci was predicted to be mainly involved in synthesizing amylosin in *B. velezensis* LS69 by blast analysis (Green box shows the core cluster of amylosin by comparative with the amylosin reported in *B. amyloliquefaciens* GA1. In *B. velezensis* FZB42 and other *B. velezensis* strains, the corresponding loci was found to encode anion permease. The flanking sequences of amylolysin loci share over 98% sequence identity in strain LS69 and other *B. velezensis* strains. The flanking sequences encode the same proteins altronate hydrolase and hypothetical protein.

Genomic islands are clusters of genes of probable horizontal origin, and they usually play an important role in antimicrobial resistance and virulence in microbes [2]. Fig. 1 shows the seven genomic islands predicted in the *Bacillus velezensis* LS69. Supplementary Table 1 provides an overview of the genes on the genetic islands. In the *B. velezensis* LS69, most of the unique genes were clustered on these islands. Fig. 2 shows the COG distribution of the core genes, accessory genes and the unique genes in 12 *B. velezensis* strains. Fig. 3 provides an extended analysis of the amylolysin cluster in *B. velezensis* LS69 and the corresponding genetic loci in other *B. velezensis* strains.

## 2 Experimental design, materials and methods

### 2.1 Genome sequencing, assembly and genetic islands prediction

The *B. velezensis* LS69 was isolated from the rice field of Lichuan city of Hubei Province (China) [1]. Whole genome sequencing produced about 1GB clean data using Illumina HiSeq. 2500 platform. A 3,917,761-bp circular chromosome was yielded by sequence assembly and gap closure. The completed genome was annotated by using NCBI Prokaryotic Genomes Automatic Annotation Pipeline (PGAAP) (https://www.ncbi.nlm.nih.gov/genome/annotation_prok/). The annotations data has been deposited in GenBank under the accession number CP015911(https://www.ncbi.nlm.nih.gov/nuccore/CP015911.1?report=gbwithparts&log$=seqview). The genetic islands of *B. velezensis* LS69 were predicted by submitting the genbank file to the IslandViewer 3 (http://www.pathogenomics.sfu.ca/islandviewer/resources/) [2]. 147 protein coding genes were clustering on the seven genetic islands.

### 2.2 Analysis of the COG distribution of core genes in 12 *B. velezensis* strains

To evaluate the evolutionary relationships of *B. velezensis* LS69, 16 *Bacillus* strains were selected to construct the *gyrA* (nucleotides 135–986 of *gyrA*) based neighbor-joining tree. The core-pan genes analysis was performed by BPGA pipeline in *B. velezensis* LS69 and the highly homologous *B. velezensis* strains [3]. These strains include the CBMB205, SQR9, FZB42, YJ11-1-4, CAU B946, JJ-D34, JS25R, NAU-B3, UCMB5033, UCMB5113 and YAU B9601-Y2. The core genome of these strains consists of 2,762 orthologous genes.

### 2.3 Prediction of the NRPS, PKS and bacteriocins gene clusters involved in synthesis of secondary metabolites

Bioinformatic tools antiSMASH [4] and BAGEL3 [5] were used to mining for the gene clusters involved in synthesizing polyketides and bacteriocins. 34 potential gene clusters were predicted in the strain LS69. Through comparative analysis with the clusters reported, ten gene clusters were found to be involved in nonribosomal synthesis of polyketides and bacteriocins. The reference gene clusters were download from the NCBI (these reference sequences are supported by corresponding papers). The accession number of the reference sequences for annotation and comparison were shown: surfactin of *Bacillus subtilis* (locus_tag of the *srfABCD*,*ycxA*,*sfp*,*yczE*: BSU03480/BSU03490/BSU03510/BSU03520/BSU03530/BSU03570/BSU03580); iturin of *Bacillus subtilis* (GenBank: AB050629.1); fengycin of *Bacillus subtilis* (GenBank accession number of the *fenABCDE*: AF023464/L42523/AF087452/AJ011849/AF023465); bacillibactin of *Bacillus subtilis* (GenBank: JQ073774.1); bacilysin of *Bacillus subtilis* (locus_tag of the *bacABCDE,ywfG*: BSU37740/BSU37730/BSU37720/BSU37710/BSU37700, BSU37690); macrolactin of *B.amyloliquefaciens* FZB42 (AJ 6340601.2); bacillaene of *B.amyloliquefaciens* FZB42 (AJ 634060.2); difficidin of *B.amyloliquefaciens* FZB42 (AJ 6340602.2); amylolysin of *Bacillus amyloliquefaciens* GA1 (KC415250.1); amylocyclicin of *B.amyloliquefaciens* FZB42 (locus_tag of the *acnBACDEF*: RBAM_029240/RBAM_029230/RBAM_029220/RBAM_029210/RBAM_029200/RBAM_029190).

### 2.4 Extended analysis of the amylolysin cluster in *B. velezensis* LS69

The amylolysin cluster was predicted by using BAGEL3, and annotated by comparative analysis with the corresponding cluster reported in *B. amyloliquefaciens* GA1 (whole genome sequence of strain GA1 has not yet been made public). Extended analysis showed the 16 kb fragment (including the amylolysin cluster) was unique for the strain LS69. This fragment was missing in the other highly homologous *B. velezensis* strains (FZB42, YJ11-1-4, CAU B946, JJ-D34, JS25R, NAU-B3, UCMB5033, UCMB5113 and YAU B9601-Y2).
