# Supplementary material for: Data on genome analysis of Bacillus velezensis LS69
Source: Data Brief. 2017 May 5;13:1–5. doi: 10.1016/j.dib.2017.04.053 (PMC5436065; doi:10.1016/j.dib.2017.04.053)
Supplement: Supplementary file 1 — Supplementary material. [file mmc2.doc]

No conflict of Interest need to be emphasized.
